# Quantification and Determination of Stability of Tylvalosin in Pig Plasma by Ultra-High Liquid Chromatography with Ultraviolet Detection

**DOI:** 10.3390/ani12111385

**Published:** 2022-05-27

**Authors:** Verónica Hernandis, Elisa Escudero, Juan Sebastián Galecio, Pedro Marín

**Affiliations:** 1 Department of Pharmacology, Faculty of Veterinary Medicine, University of Murcia, 30100 Murcia, Spain; escudero@um.es (E.E.); or jsgalecio@um.es (J.S.G.); pmarin@um.es (P.M.); 2 Escuela de Medicina Veterinaria, Colegio de Ciencias de la Salud, Universidad San Francisco de Quito, Cumbayá 170157, Ecuador

**Keywords:** pigs, HPLC, plasma, tylvalosin

## Abstract

**Simple Summary:**

Tylvalosin (TV) is a third-generation macrolide antibiotic, registered exclusively for veterinary medicine to treat respiratory and enteric bacterial infections in swine and poultry. In the coming years, the use of this drug will probably be widely studied in different species. However, before its use in each veterinary species, macrolide analytical determination in various biological fluids is a pre-requisite step for the rational dose calculation of TV based on specific pharmacokinetic information. Its quantification is essential to detect and avoid the appearance of residues in animal products intended for human consumption. Therefore, the aim of this proposed method was to develop a high-performance liquid chromatography (HPLC) method with ultraviolet detection for TV quantification in pig plasma. According to the food and drug administration (FDA) guidelines, this quick, sensitive, and reproducible validated method produced a good performance. This method can be useful for storing plasma samples for months and for performing routine analysis and pharmacokinetic studies.

**Abstract:**

Tylvalosin (TV) is a macrolide antibiotic that is used for treating respiratory and enteric bacterial infections in swine and in poultry. In the coming years, the use of this drug will probably be widely studied in different species, but before its use in each veterinary species, macrolide analytical determination in various biological fluids is a pre-requisite step for the rational dose calculation of TV based on specific pharmacokinetic information. Its quantification is essential for detecting and avoiding the appearance of residues in animal products intended for human consumption. Therefore, a robust chromatographic method coupled with an ultraviolet detector was fully validated for the quantification of TV in pig plasma. A mixture (78:22) of (A) 0.3% formic acid in water and (B) acetonitrile was used as the mobile phase. TV and enrofloxacin (internal standard) were eluted at 14.1 and 5.9 min, respectively. Calibration curves ranged from 0.1 to 5 μg/mL. The accuracy and precision parameters for the quality controls were always <13.0%. Recovery ranged from 89.66 to 96.92%. The detection and quantification limits were found to be 0.05 μg/mL and 0.1 μg/mL, respectively. This method could be applied to develop pharmacokinetic studies.

## 1. Introduction

Tylvalosin (TV) is a modern veterinary macrolide antibiotic with a 16-membered lactone ring derived from tylosin by the substitution of the 3-acetyl-40-isovaleryl group for acetylisovaleryltylosin tartrate [1,2]. This macrolide is used in swine and poultry for treating enteric and respiratory bacterial infections, including those caused by *Mycoplasma* species [1,3,4]. Before its use in veterinary medicine, macrolide analysis in body fluids is a prerequisite step for the rational dose calculation of TV therapy based on specific pharmacokinetic information in veterinary species. Moreover, its quantification is essential to detect and avoid the appearance of residues in animal products intended for human consumption. Initially, microbiological methods were used to quantify the TV plasma concentrations in broiler chickens and turkeys [5,6,7]. These methods can measure antibiotic concentrations, assessing their inhibitory effects on the growth of specific test microorganisms. Microbiological assays are a potential alternative to high-performance liquid chromatography (HPLC) methods that can unmask undetectable changes not checkable by conventional chemical methods, requiring no specific equipment and with non-toxic products [1]. However, HPLC methods, although more laborious, are more accurate, reproducible and precise than the aforementioned, being appropriate for pharmacokinetic studies. Therefore, liquid chromatography coupled with mass spectrometry (HPLC/MS) techniques have been widely used in previous studies to quantify TV in feed, milk, honey, tissues and eggs [8,9,10,11,12,13].

Nowadays, only three methods can be found in the literature to determinate TV in plasma by HPLC. One of these methods quantifies the concentrations of this macrolide by liquid chromatography–mass spectrometry (LC/MS) in pig plasma with a quantification limit (LOQ) of 0.001 µg/mL [14]; the other two methods use high-performance liquid chromatography with ultraviolet detection (HPLC/UV) in turkey plasma and chicken serum [4,15] with an LOQ of 0.1 and 0.039 µg/mL, respectively. LC/MS methods show high sensitivity but unfortunately suffer from a high matrix effect (especially in mode: electrospray ionization), and these devices are not usually present in most clinical laboratories [16,17,18]. Therefore, other chromatographic methods with sufficient sensitivity and reliability to be implemented in routine analyses ought to be developed.

Regarding sample preparation, different methods have been used, such as solid-phase extraction (SPE) [14] and liquid–liquid extraction (LLE) [4,15]. Solid-phase extraction is widely used in liquid chromatography (LC) as high recoveries and good chromatograms are obtained, but it takes longer to perform and requires more steps [19,20,21]. On the other hand, protein precipitation (PP) is the simplest method to remove proteins from plasma samples [22], and for that reason, researchers are developing new and easier PP methods, instead of SPE or LLE, which have long running times. Owing to the lack of suitable data and stability tests of TV in pig plasma samples, it is necessary to develop an easy, rapid, cost-effective and eco-friendly chromatographic method with sufficient sensitivity and reliability to be applied when LC/MS is not available in the laboratory. The aim of this research is therefore to achieve a rapid, eco-friendly and reliable method to quantify TV in pig plasma by LC with an ultraviolet detector.

## 2. Materials and Methods

### 2.1. Reagents and Materials

Tylvalosin (TV) tartrate (TR-T634280), enrofloxacin (internal standard, IS) and other substances examined to select IS and check specificity (difloxacin, marbofloxacin, norfloxacin, ciprofloxacin, penicillin G, streptomycin, cefuroxime, cephazolin, cephadroxil, tildipirosin, tylosin, ceftiofur hydrochloride, pregnenolone, alfaxalone and candesartan) were obtained from Cymit Química (Barcelona, Spain). Acetonitrile (ACN-HPLC analytical grade), formic acid (98–100%), dimethyl sulfoxide (DMSO) and water were obtained from Merck Life Science (Madrid, Spain). Potassium di-hydrogen phosphate 99%, di-potassium hydrogen phosphate 99%, and sodium hydroxide pearls of 98% purity were purchased from PanReac AppliChem (Barcelona, Spain).

For method application, tylvalosin (Aivlosin^®^) was obtained as 62.5% water-soluble white granules (ECO Animal Health Europe Limited, Dublin, Ireland). Each gram of powder contains 625 mg of TV as TV tartrate.

### 2.2. Instrumentation and Analytical Conditions

The liquid chromatography (LC) system, column and guard column were described in a previous paper [23].

The mobile phase was composed of 0.3% formic acid in aqueous phase acetonitrile (78:22), eluted in an isocratic manner at 1.0 mL/min and at 22 °C. A volume of 50 µL was injected. A wavelength of 254 nm was chosen in the ultraviolet detector. TV and enrofloxacin in pig plasma were eluted at 14.1 min and 5.9 min, respectively, and the total runtime was 15 min.

### 2.3. Standard Solutions

TV and enrofloxacin (IS) stock solutions were prepared at 100 µg/mL and 25 µg/mL, respectively. Solutions of TV and IS were made separately by dissolving each compound in 2 mL of DMSO, and a solution of phosphate buffer 0.2 M (pH = 7.4) was added into two 100 mL volumetric flasks. Stock solutions were further diluted with 0.2 M of phosphate buffer (pH = 7.4) to prepare working solutions at low concentrations. Working solutions of TV at 2.5, 5, 10, 25 and 50 µg/mL were prepared daily.

### 2.4. Calibration Curves and Quality Control Samples

Plasma drug-free samples were obtained from the slaughterhouse (Murcia, Spain). After stunning, blood samples were collected from the jugular veins in heparinized tubes and were centrifugated at 1500 g for 15 min. Plasma was immediately removed and stored at −40 °C until use. The Bioethical Committee of the University of Murcia (Spain) approved the experimental protocol (CEEA 558/2019) before the study. Calibration curves (CC) and quality control (QCs) samples were set up according to food and drug administration (FDA) guidelines [24]. Then, 500 µL of blank pig plasma was spiked with a proper quantity of TV working solution. After mixing, 20 µL of IS (25 µg/mL) were added to these samples. Nine concentration levels were used to compose the plasma calibration curves: 0.1, 0.25, 0.5, 0.75, 1, 1.5, 2, 3 and 5 µg/mL. For QCs, four concentration levels were used (0.1, 0.5, 1 and 2 μg/mL).

### 2.5. Sample Preparation

Aliquots of plasma (500 μL) were fortified with 20 µL of IS (25 µg/mL). After mixing, 400 µL of mixture (0.1% formic acid in acetonitrile) was added. This solution was mixed in a vortex for 1 min and then shaken for 5 min in an ultrasonic bath at room temperature. Afterwards, the solution was centrifuged at 15,000 g for 10 min. Subsequently, the upper phase was transferred to a polypropylene tube until it was dry due to evaporation. A volume of 75 μL of water was added to the residue, which was then transferred to high-performance liquid chromatography (HPLC) vials.

### 2.6. Method Validation

The present method was performed and validated following FDA guidelines for bioanalytical method validation [24] to quantify TV in pig plasma. Therefore, all relevant parameters mentioned in the guidance were evaluated.

### 2.7. Method Application

To explore the applicability of the method in the clinical practice, TV concentrations were measured in plasma samples obtained from two cross bred pigs weighing approximately 20 kg. TV was given as a single dose at 5 mg/kg (according to the manufacturer instructions). TV water soluble granules (ECO Animal Health Europe Limited, Dublin, Ireland) was dissolved in water and administered in a single bolus by oral gavage.

Blood samples were collected from the jugular veins into heparinized tubes at 2, 4 and 24 h. Samples were centrifuged at 600× *g* for 10 min, and plasma was stored at −40 °C until analysis. Plasma samples (500 μL) were spiked with 20 µL IS before analysis. The Bioethical Committee of the University of Murcia (Spain) approved the experimental protocol (CEEA 558/2019).

## 3. Results

### 3.1. Linearity, Detection and Quantification Limits

**Figure 1:**
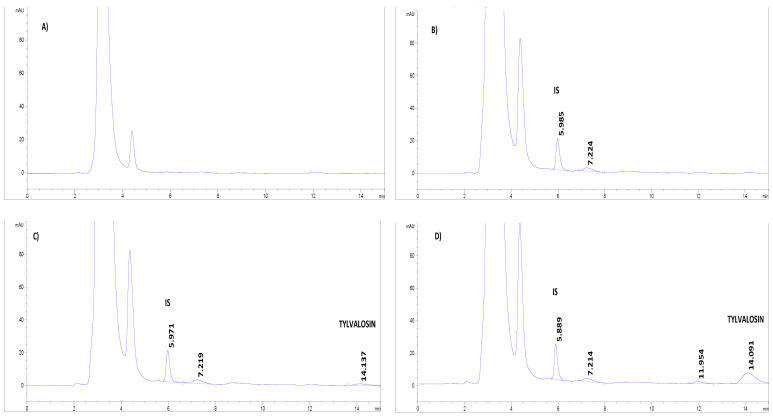
Chromatograms of tylvalosin and enrofloxacin (IS) in pig plasma by HPLC-UV. (**A**) Blank plasma; (**B**) blank plasma with 20 µL of IS (25 µg/mL); (**C**) blank plasma with tylvalosin at LOQ (0.1 µg/mL) and 20 µL of IS (25 µg/mL); and (**D**) blank plasma spiked with tylvalosin (1 µg/mL) and 20 µL of IS (25 µg/mL).

Tylvalosin (TV) and internal standard (IS) peaks in pig plasma were eluted at 14.1 min and 5.9 min, respectively (Figure 1).

**Figure 2:**
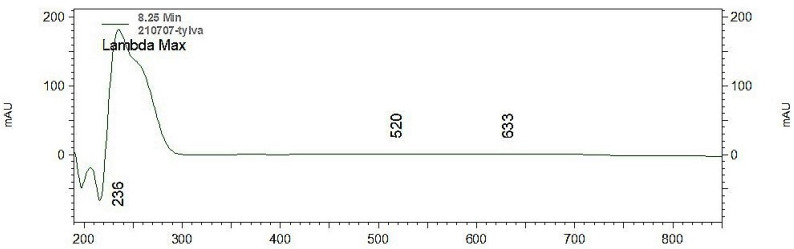
Absorption spectrum of tylvalosin standard.

Tylvalosin was analyzed by high-performance liquid chromatography with diode-array detection (HPLC-DAD) (Agilent, Madrid, Spain) at different wavelengths (220, 254, 280 and 420 nm) to identify the best one. A higher area of TV was obtained at 220 nm. Moreover, the ultraviolet (UV) spectrum in the DAD detector was obtained (Figure 2), and the best wavelength was found at 236 nm.

**Figure 3:**
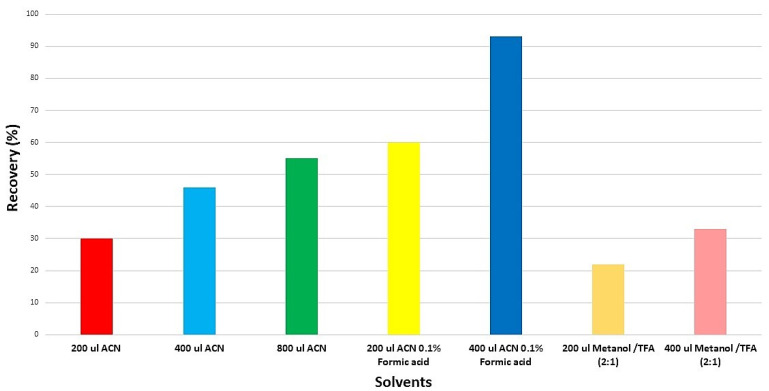
Recovery of tylvalosin (500 µg/mL of pig plasma) after adding different microliters of solvents as the extracted reagent.

Different solvents were proposed to extract tylvalosin from the pig plasma samples (Figure 3). Acetonitrile with 0.1% formic acid was found to be the best option.

To evaluate the linearity, calibration curves were established following a previously described method [23]. The linearity ranged from 0.1 µg/mL to 5 µg/mL and was described by the equation y = 0.9993x + 0.0021 (r^2^ ≥ 0.9998). The detection (LOD) and quantitation limits (LOQ) were 0.05 and 0.1 µg/mL, respectively, which is sufficiently sensitive for the quantification of TV.

### 3.2. Precision and Accuracy

Food and drug administration (FDA) guidance [24] was followed to calculate the precision and accuracy. The results are shown in Table 1. Four levels with replicates (*n* = 5) were analyzed for intra-day and inter-day quality control assays. Both the intra-day and inter-day precision values were <6.1%. The intra-day accuracy was within the range from −7.96 to 12.50%. The inter-day accuracy varied from −9.05 to 12.89%. For the precision and accuracy acceptance criteria, the results should meet ≤15% coefficient of variation (CV). Relevant results were obtained, indicating that this method is adequate for quantifying TV in pig plasma.

### 3.3. Recovery

The recovery results are shown in Table 2. Tylvalosin was analyzed according to the FDA recovery test [24]. A brief explanation can be found in [23]. The values ranged from 89.66 to 96.92% with a variability (CV) of <7.75%. Excellent recoveries close to 100% indicate an efficient and reproducible method of quantifying TV in pig plasma samples.

### 3.4. Selectivity, Specificity, Dilution Effect and Carry Over

No interfering peaks or endogenous interferences were observed at the same retention times as TV and IS (Figure 1). In addition to this, an appropriate retention time and symmetric sharp for TV and IS were found. Other drugs (difloxacin, marbofloxacin, norfloxacin, ciprofloxacin, penicillin G, streptomycin, cefuroxime, cephazolin, cephadroxil, tildipirosin, ceftiofur hydrochloride, pregnenolone, alfaxalone and candesartan) were analyzed in order to demonstrate the specificity of the method. The method is appropriate for the analysis of TV in pig samples. To verify the applicability of the method proposed here, a dilution effect integrity test was also performed to ensure the accuracy of the measurements when the samples were diluted. Concentrations of 1 and 5 µg/mL in pig plasma were prepared separately and diluted 10-fold with matrix. The accuracy results were less than ±15% of the nominal concentration, and the precision (%CV) was within 15%. Moreover, carryover was tested in blank plasma, after the injection of a high concentration of TV (5 µg/mL (*n* = 6)), resulting in a signal <20% of the LOQ. These data suggest the absence of a significant carryover effect.

### 3.5. Stability

The results of the stability assessment of TV in quality controls and stock solutions are shown in Table 3 and Table 4, respectively. For quality control (QCs) samples, at 4 °C, room temperature and 24 h were evaluated in terms of short-term stability according to FDA guidelines [24]. They were stable, with CV < 5.9%, and bias values were always ≤11.0%. The stability results of TV in QCs were suitable when they were stored for 15 and 30 days at −40 °C. At 15 days, a low QC (0.5 µg/mL) accuracy of −8.73 (CV 6.26%) was found, and at 30 days, it was −2.58% (CV 6.61%). Likewise, a degradation of −8.51% for 15 days (CV 0.94%) and −9.77% (CV 2.83%) for 30 days of storage were obtained for the high QC (2.0 µg/mL). The degradation of QCs at 30 days is greater than at 15 days, as was to be expected. In addition to this, the freeze–thaw stability (five cycles at −40 °C) was analyzed, and the TV concentration only decreased by 3.31% (CV 10.79%) for a QC of 0.5 µg/mL and by 10.76% (CV 3.87%) for a QC of 2.0 µg/mL. At the same time, 2.5 µg/mL and 25 µg/mL stock solutions were stored for 7, 20 and 60 days at −40 °C, achieving good stability test results (Table 4). In this case, a slight decrease of <4.0% for 7, 20 and 60 days was observed, indicating that stock solutions of TV can be stored for up to 60 days.

### 3.6. Method Application

**Figure 4:**
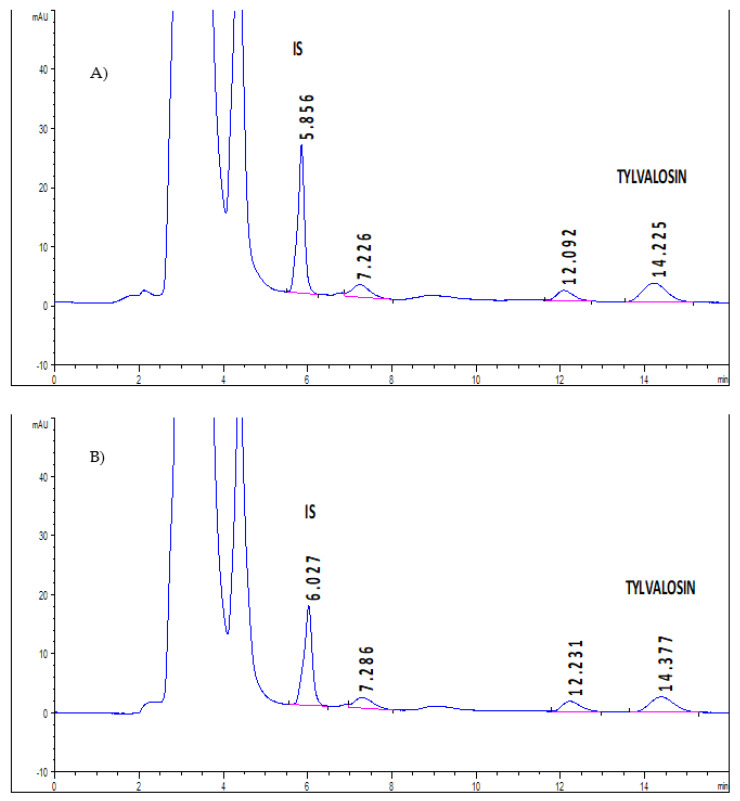
Representative HPLC chromatogram of pig plasma samples after oral TV administration (5 mg/kg) at time sampling: (**A**) 2 h and (**B**) 4 h.

The application of the method was tested by analyzing plasma samples obtained from two pigs after TV oral administration. The drug was detected in all samples without interferences, as shown in Figure 4.

## 4. Discussion

### 4.1. Optimization of Chromatographic Conditions

An analytical method of validation to quantify tylvalosin (TV) in pig plasma by liquid chromatography (LC) with an ultraviolet detector has not yet been published. Only three methods have been reported to quantify TV in plasma, one of them using HPLC/MS [14] and the others using high-performance liquid chromatography with ultraviolet detection (HPLC/UV) (turkey plasma and chicken serum) [4,15]. Differences have been reported among different species in terms of the composition and functional properties of blood. Higher levels of hemoglobin and plasma protein content were found in porcine blood as compared with chicken and duck blood. The porcine blood seemed to have a less nutritive amino acid composition than avian blood due to a larger deficiency in isoleucine, but it was rich in heme iron content compared with avian blood, in relation to its higher hemoglobin content. Moreover, chicken blood exhibited superior gelation and emulsion properties but poor foaming properties as compared with duck and porcine blood [25,26]. Therefore, these differences could influence the extraction ratio of plasma samples and different endogenous peaks in the chromatograms; in fact, any analytical determination of the biological matrix must be validated in the specific matrix.

The main advantages of our HPLC/UV method are that it reduces costs and time in the analytical process in accordance with the principles of green chemistry [27,28], with our method showing the following differences with the HPLC/UV methods previously published in avian species [4,15]: a lower consumption of organic solvents (acetonitrile: 22% in the pig method, 51% in both avian methods), and a cheaper column and filters, notably reducing the price and time of the analysis. Moreover, our method has a short running time (15 min), although this cannot be compared between methods because the retention times of the drugs (analyte and internal standard) and the duration of the chromatograms have not been published for both avian methods [4,15]. Therefore, an eco-friendly method was developed following some principles of green chemistry, such as the minimization of energy consumption, waste chemicals and material amounts, easier sample preparation and the maximization of sample throughput.

Tylvalosin is a derivate of tylosin and could potentially be detected at the same wavelength. At first, when using the chromatographic method previously published by our group to quantify tildipirosin [29] (using tylosin as internal standard), the results were not satisfactory because the wavelength (289 nm) was not appropriate to quantify TV with acceptable precision and accuracy at the quantification limit (LOQ). However, only one article has quantified TV at 289 nm [4]. Other methods have reported wavelengths between 280 and 287 nm to detect tylosin in different matrices by HPLC/UV with an acceptable LOQ [30,31,32,33]. For this reason, the maximum wavelength of TV absorption was examined. Tylvalosin was analyzed by high-performance liquid chromatography with diode-array detection (HPLC-DAD) (Agilent, Madrid, Spain), and various wavelengths were tested (220, 254, 280 and 420 nm) to identify the best one. Surprisingly, a higher area of TV was obtained at 220 nm. Moreover, the UV spectrum in the DAD detector was obtained (Figure 2), and the best wavelength was found at 236 nm. Based on these results, the detector was fixed at 220 and 236 nm, but it was not very stable, and therefore, λ = 254 nm was finally chosen as the wavelength of the UV detector.

Concerning the mobile phases, buffer solutions could cause problems in the LC system, such as clogging in the pump; an acidic mobile phase is one of the best ways to avoid these problems. The current trend in green chemistry is to avoid the use of buffers [34,35,36]. Moreover, isocratic elution was selected with a short running time instead of gradient elution.

Several C18 columns available in the laboratory were also checked in [23]. The Brisa LC2 C18 column 250 mm × 4.6 mm i.d, 5 µm, was chosen as it possessed the best symmetrical peaks.

Concerning sample preparation, protein precipitation (PP) and liquid–liquid extraction (LLE) were tested. The precipitation of protein was chosen because it is fast and eco-friendly [19,22]. Different ratios of acetonitrile added to a small quantity of acid or a mixture of methanol/trifluoroacetic acid were checked to obtain the best recoveries (Figure 3). Finally, acetonitrile with 0.1% formic acid was found to be the best option.

### 4.2. Validation

In general, previously reported validation methods to quantify TV in plasma are not described in detail because they are part of a pharmacokinetic study. Owing to the lack of suitable data in pig plasma samples, it is important to develop an easy, cost-effective and eco-friendly chromatographic method.

The LOQ value obtained in this study was 0.1 µg/mL. This result is similar to those obtained by other authors from avian plasma samples of 0.1 and 0.039 µg/mL [4,15]. A lower LOQ (0.001 µg/mL) was obtained in pig plasma [14] when TV was quantified by liquid chromatography–mass spectrometry (LC/MS) with solid-phase extraction (SPE). It must be admitted that high-performance liquid chromatography–mass spectrometry (HPLC/MS) methods have higher sensitivity than HPLC-UV and high-performance liquid chromatography with fluorescence detection (HPLC-FL) methods, but SPE, commonly used in mass spectrometry, results in longer analytical times per sample compared with the presented HPLC/UV method with protein precipitation.

Furthermore, the intra-day and inter-day precision and accuracy values for quality control (QCs) samples were not reported in most previous manuscripts. Only intra-day and inter-day precision coefficient of variation (CV) values, ranging from 4.28 to 4.92% and from 4.86 to 5.42%, respectively, were obtained for broiler chicken and turkey plasma [4]. On the other hand, these parameters were not evaluated in synovial fluid and pig plasma [14,37]. Therefore, the precision and accuracy values of the present method (Table 1) have been proven to be able to quantify TV in pig plasma in accordance with the established standards [28].

The mean recovery value was 93.87%, which was higher than the results obtained in broiler chickens (81–83%) [15] and turkeys (87.2%) [4]. The mean recoveries for synovial fluid and pig plasma were not reported [14,37] nor were selectivity, specificity and carry-over results [4,14,37]. Tylvalosin stability in synovial fluid was checked at −70 °C for 7, 14, and 28 days [37]. The author ensured stability at −70 °C, but the results are not shown. Results to measure stability in QCs and stock solutions are shown in Table 3 and Table 4, respectively. These results demonstrate stability at −40 °C, supporting TV stability at −70 °C [14]. In brief, our findings demonstrate a consistent analytical method to quantify TV with accuracy and speed; as TV was analyzed in isocratic mode, the chromatogram run times were short (15 min) and a simple PP procedure was used for sample preparation. Moreover, the calibration curves were linear over a concentration range of 0.1–5 μg/mL, and the limit of quantification was good (0.1 μg/mL) due to an excellent recovery result (93.52%).

## 5. Conclusions

The present chromatographic method is easy to use due to its simplicity. The parameters were validated according to food and drug administration (FDA) guidance. The tylvalosin plasma samples and stock solutions can be stored for a long time with the utmost safety. Overall, this method can be applied to perform pharmacokinetic studies.

## Figures and Tables

**Table 1:** Intra- and inter-day accuracy (bias) and precision (CV) of tylvalosin in pig plasma (*n* = 5).

	Intra-Day (*n* = 5)			Inter-Day (*n* = 5)		
Concentration (µg/mL)	Mean ± SD (µg/mL)	CV (%)	Bias (%)	Mean ± SD (µg/mL)	CV (%)	Bias (%)
0.1	0.11 ± 0.01	1.23	12.50	0.10 ± 0.01	5.82	2.96
0.5	0.49 ± 0.02	4.81	−2.10	0.56 ± 0.01	0.88	12.89
1	0.89 ± 0.04	4.02	−10.99	1.09 ± 0.03	3.04	−9.05
2	1.84 ± 0.11	6.04	−7.96	2.09 ± 0.06	2.99	4.83

**Table 2:** Recovery of tylvalosin in pig plasma (*n* = 5 replicates).

	Recovery (%)	
Concentration (µg/mL)	Mean ± SD	CV (%)
0.5	89.66 ± 6.93	7.73
1	95.02 ± 6.77	7.12
2	96.92 ± 4.65	4.80

**Table 3:** Short-term, long-term and freeze–thaw stability of quality controls (QCs) of tylvalosin (*n* = 5 replicates).

Condition		QC Low 0.5 (µg/mL)			QC High 2.0 (µg/mL)		
		Mean ± SD (µg/mL)	CV (%)	Bias (%)	Mean ± SD (µg/mL)	CV (%)	Bias (%)
Short-term stability	0 h	0.47 ± 0.03	5.86	−4.91	2.05 ± 0.05	2.52	2.83
	24 h (RT)	0.44 ± 0.02	3.9	−11.00	1.83 ± 0.10	5.53	−8.37
	24 h (4 °C)	0.44 ± 0.01	2.99	−10.54	1.86 ± 0.04	2.44	−7.17
Long-term stability (−40 °C)	15 days (−40 °C)	0.45 ± 0.03	6.26	−8.73	1.83 ± 0.02	0.94	−8.51
	30 days (−40 °C)	0.48 ± 0.03	6.61	−2.58	1.80 ± 0.05	2.83	−9.77
5 Freeze–thaw cycles (−40 °C)	5 days of freeze–thaw, (−40 °C)	0.48 ± 0.05	10.79	−3.31	1.78 ± 0.07	3.87	−10.76

**Table 4:** Short- and long-term stability of stock solutions of tylvalosin (*n* = 5 replicates).

Condition		Stock Solution Low 2.5 (µg/mL)			Stock Solution High 25 (µg/mL)		
		Mean ± SD (µg/mL)	CV (%)	Bias (%)	Mean ± SD (µg/mL)	CV (%)	Bias (%)
Short-term stability	0 h	2.45 ± 0.16	6.59	−2.13	25.20 ± 1.20	4.76	0.81
	7 days (−40 °C)	2.59 ± 0.01	0.19	3.89	24.18 ± 0.16	0.63	−3.28
Long-term stability (−40 °C)	20 days (−40 °C)	2.53 ± 0.14	5.56	1.22	24.73 ± 1.44	4.63	−1.07
	60 days (−40 °C)	2.41 ± 0.23	9.41	−3.67	24.54 ± 0.32	1.29	−1.82

## Data Availability

Data is contained within the article.
